# Lipidomic insights on abdominal aortic aneurysm and peripheral arterial disease

**DOI:** 10.1007/s00109-025-02524-1

**Published:** 2025-02-26

**Authors:** Helena Beatriz Ferreira, Fábio Trindade, Rita Nogueira-Ferreira, Adelino Leite-Moreira, Rita Ferreira, Marina Dias-Neto, M. Rosário Domingues

**Affiliations:** 1 Mass Spectrometry Center, LAQV-REQUIMTE, Department of Chemistry, University of Aveiro, Campus Universitário de Santiago, 3810-193 Aveiro, Portugal; 2 RISE-Health, Department of Surgery and Physiology, Faculty of Medicine, University of Porto, 4200-319 Porto, Portugal; 3 Department of Cardiothoracic Surgery, Centro Hospitalar Universitário São João, 4200-319 Porto, Portugal; 4 Department of Angiology and Vascular Surgery, Unidade Local de Saúde São João, Porto, Portugal; 5 CESAM – Centre for Environmental and Marine Studies, Department of Chemistry, University of Aveiro, Campus Universitário de Santiago, 3810-193 Aveiro, Portugal

**Keywords:** Abdominal aortic aneurysm, Peripheral arterial disease, Cardiovascular diseases, Lipidomics, Mass spectrometry

## Abstract

Abdominal aortic aneurysm (AAA) and peripheral arterial disease (PAD) are two cardiovascular diseases associated with considerable morbidity, mortality and quality of life impairment. As they are multifactorial diseases, several factors contribute to their pathogenesis, including oxidative stress and lipid peroxidation, and these may have key roles in the development of these pathologies. Alterations of the lipid metabolism and lipid profile have been reported in cardiovascular diseases but to a lesser extent in AAA and PAD. Modifications in the profile of some molecular lipid species, in particular, native phospholipid and triglyceride species were mainly reported for AAA, while alterations in the fatty acid profile were noticed in the case of PAD. Oxidized phospholipids were also reported for AAA. Although AAA and PAD have a common atherosclerotic root, lipidomics demonstrates the existence of distinct lipid. Lipidomic research regarding AAA and PAD is still scarce and should be set in motion to increase the knowledge on the lipid changes that occur in these diseases, contributing not only to the discovery of new biomarkers for diagnosis and prognosis assessment but also to tailor precision medicine in the clinical field.

## Introduction

Cardiovascular diseases (CVD) are a vast group of disorders that affect the heart and blood vessels and are the main causes of death globally. Besides the high rate of mortality, CVD are also accountable for the economic burden regarding health care, both in hospitals and at home (informal care) [1]. The prominent impact of CVD on nowadays’ society is essentially attributed to its asymptomatic nature during the “silent” onset and progress of plaque deposition and/or vessel dilation over time, respectively, leading to blood vessel obstruction or rupture [2].

Lipids and, in particular, oxidized lipids are important players in the formation of atherosclerotic plaque and in the underlying inflammatory process. Therefore, they are recognized as significant players in the pathophysiology of CVD, and thus, lipids are considered promising biomarkers of CVD. As demonstrated in a few studies that report lipid alterations in CVD (gathered in an elegant review [2]), lipids can provide specificity for several conditions; however, most studies are centred on general atherosclerosis and coronary heart disease (including myocardial infarction). Hence, the present review will focus on abdominal aortic aneurysm (AAA) and peripheral arterial disease (PAD). These two pathologies are associated with considerable morbidity, mortality and quality of life impairment and, unfortunately, were forgotten during the COVID-19 pandemic [3]. Many follow-up visits to monitor the development of both AAA and PAD were lost due to COVID-19 limitations; thus, their medical management is currently suboptimal, possibly leading to an increased incidence and major impact of these diseases in the near future.

Both AAA and PAD share fundamental similarities in their vascular pathology. They both involve structural and functional changes in blood vessels, resulting in compromised blood flow and potential complications. Atherosclerosis stands out as a key contributor to both conditions, fostering the development of arterial wall lesions that can lead to stenosis and/or aneurysm formation [4, 5]. One notable aspect common to AAA and PAD is their tendency to progress silently, particularly in their early stages [6–8]. This emphasizes the importance of early detection and screening efforts to prevent adverse outcomes [9]. However, despite these parallels, they exhibit clear differences in anatomical localization, risk factors and clinical presentations [10, 11].

PAD, with a higher global incidence affecting over 110 million people worldwide, predominantly manifests as stenosis or occlusion of arteries supplying various anatomical regions, particularly the lower limbs [4, 12, 13]. In contrast, though less prevalent, AAA remains a significant health concern, especially among older adults [14]. It primarily affects the abdominal aorta, leading to a localized expansion [5]. Symptomatology also varies between the two conditions. AAA often remains asymptomatic until rupture, which can have lethal consequences [9]. Symptoms, if present, may include back or abdominal pain [8]. Conversely, PAD commonly presents with symptoms such as intermittent claudication, i.e. recurrent fatigue, cramping or pain in the lower extremities, reflecting a compromised blood flow to the affected areas [11]. Moreover, while both conditions share common risk factors such as age, hypertension and smoking, diabetes exhibits a nuanced relationship. It appears to have a negative association with AAA [15] but poses an elevated risk for PAD development [16]. Additionally, male sex emerges as a significant risk factor for AAA [17].

Despite the multitude of factors contributing to the increased risk and progression of AAA and PAD, diagnosing and making the prognosis of these conditions remain challenging due to the absence of specific biomarkers, especially in the presence of other atherosclerotic diseases. Like many other inflammatory disorders, both AAA and PAD are intricately linked to oxidative stress and lipid peroxidation, as explored in subsequent sections. These pathways are directly involved in the pathogenesis of these vascular diseases, offering potential avenues for improved diagnostic accuracy and targeted therapeutic interventions.

## Oxidative stress and lipid dynamics in AAA and PAD

Vascular wall inflammation is a hallmark of AAA and PAD and is characterized by significant and increased production of inflammatory molecules and reactive oxygen species (ROS) [14, 18–20]. In fact, inflammation goes hand in hand with oxidative stress since they promote each other in a vicious cycle. The inflammatory process releases pro-inflammatory cytokines, which promote lipid and protein oxidation in artery walls, potentiating an oxidative environment with consequent tissue injury [21]. Besides inflammation, oxidative stress can be further exponentiated by either the increase of ROS production or the decrease/progressive dysfunction of the antioxidant defence system. Either way, it leads to alterations in the activity of enzymatic/non-enzymatic antioxidants and in lipid peroxidation species [21].

For both AAA and PAD, the endogenous antioxidant defences were shown to be dysregulated (Table 1). On the one hand, the activity of the enzymes Cu/Zn superoxide dismutase, Mn superoxide dismutase, glutathione reductase and glutathione peroxidase was found to be lower in aortic tissue samples [22] infra-renal aortic biopsies [23] and in serum [24] from patients with AAA. In the same line, a lower activity of paraoxonase-1 in serum was associated with endothelial dysfunction in symptomatic PAD patients [25]. On the other hand, NADPH oxidase activity and the levels of O_2_^●−^ were found to be increased in human aneurysmal aortic biopsies [26]. Nrf2/Heme-oxygenase 1 and glutathione-SH levels were found to be significantly decreased in the plasma of PAD patients [27].

**Table 1:** Changes induced by oxidative stress in AAA and PAD

Vascular disease	Oxidative changes	
	↓ Decrease	↑ Increase
AAA	Cu/Zn superoxide dismutase [22–24] Mn superoxide dismutase [22–24] Glutathione reductase [22–24] Glutathione peroxidase [22–24]	ROS [14, 18–20], free radical O_2_^•−^ [26] NADPH oxidase [26], COX-2 levels [28–30] Leukotrienes [31, 32], prostaglandin E2 [28–30] MDA (↑↑in ruptured aneurysm) [23, 24] Lipid hydroperoxides (↑↑in ruptured aneurysm) [23, 24]
PAD	Paraoxonase-1 [25] Nrf2/Heme-oxygenase 1 [27] Glutathione-SH [27]	ROS [14, 18–20] MDA [19, 33–36] F2-isoprostanes [19, 33–36] 4-HNE [37, 38]

*AAA* abdominal aortic aneurysm, *PAD* peripheral arterial disease, *Cu/Zn* copper/zinc, *Mn* manganese, *Glutathione-SH* reduced glutathione, *ROS* reactive oxygen species, *NADPH* reduced nicotinamide adenine dinucleotide phosphate, *COX-2* cyclooxygenase 2, *MDA* malondialdehyde, *4-HNE* 4-hydroxynonenal

The efficacy of antioxidant therapies was explored as well; however, the reported results for AAA concern only mouse models and do not include human trials. Nonetheless, folic acid, vitamin C, vitamin E, ß-carotene, resveratrol and quercetin were reported to inhibit ROS production in AAA models [39–44]. Moreover, glutathione-SH, vitamin C, vitamin E and α-tocopherol supplementation were proven helpful in combating oxidative damage in PAD patients, improving several prognostic parameters [45–50].

Oxidative stress promotes lipid peroxidation, a common finding in AAA and PAD (Table 1). In a general manner, lipoxygenase (LOX) and cyclooxygenase (COX) are two enzymes responsible for the oxidation of lipids, either by catalyzing the oxygenation of polyunsaturated fatty acids (PUFA) [51] or by promoting the conversion of arachidonic acid (AA) into prostaglandins [52], respectively. These enzymes play a role in the generation of ROS, thereby exacerbating oxidative stress, which could also be involved in vascular changes [53]. Leukotrienes, which are key inflammatory mediators, stem from LOX activity and have been reported to be increased in aneurysmal aortas [31, 32]. This suggests a potential role for LOX in AAA pathogenesis. Additionally, COX-2 has been associated with increased aortic remodelling [54], as evidenced by elevated levels of COX-2 and its metabolite, prostaglandin E2, within aneurysmal tissue [28–30]. While COX inhibitors could be regarded as suitable for limiting AAA development, concerns arise regarding their association with increased incidence of cardiovascular events [55].

Lipid peroxidation has been associated with apoptosis and necrosis of aortic cells [56, 57], potentially paving the way for PAD, aortic weakening and AAA development. The levels of the lipid peroxidation products malondialdehyde and lipid hydroperoxides were found to be increased in both atherosclerotic aortic tissue and sera of AAA patients, particularly those with ruptured aneurysms [23, 24]. In the context of PAD, plasma levels of malondialdehyde and F2-isoprostanes were found significantly higher in patients compared to controls [19, 33–36]. Furthermore, protein adducts with 4-hydroxynonenal (4-HNE), a well-known secondary product of lipid peroxidation, have been found in smooth muscle cells and cardiac muscle of PAD patients, with the accumulation of 4-HNE emerging as a marker for oxidative stress-induced muscle cell injury [37, 38].

The oxidized phospholipids can be degraded by phospholipase A2 and generate lyso-phospholipids. In addition, the synthesis of lipid mediators by enzymatic oxidation of fatty acids, which are released from the cellular membrane, may also lead to the formation of lyso-lipids [58, 59]. Thus, all these alterations may lead to changes in the homeostasis/metabolism and in the lipid profile. Therefore, understanding the intricate alterations in the lipidome of tissues, particularly vessel cells, as well as in circulation, resulting from lipid peroxidation in AAA and PAD is paramount for unravelling disease mechanisms and pinpointing potential therapeutic targets.

## Lipidomics advancing knowledge of AAA and PAD pathogenesis

The current clinical, biochemical and imaging parameters to monitor the progression of PAD and AAA exhibit limited predictive value and are poorly efficient. Thus, the identification of specific molecular markers able to provide both an early diagnosis during the asymptomatic stages of the disease and monitorization of disease progression after surgical intervention is imperative. Given the multifactorial nature of AAA and PAD, single biomarker determination may not fully capture the complex pathophysiological processes underlying vascular remodelling [60]. Therefore, adopting a multimarker panel could aid clinicians in decision-making regarding the management of AAA and PAD. Given the association of dyslipidaemia and altered lipid metabolism with these diseases, circulating lipids emerge as valuable targets in the quest for a molecular signature suitable for assessing and managing these conditions in clinical settings [2].

High-throughput lipidomics enables the detection of lipid variations at a molecular level, providing valuable insights into several chronic diseases [61]. Presently, clinical lipidomics has emerged as a reliable and consistent approach for understanding and identifying lipid diagnostic biomarkers and lipid therapeutic targets [62].

In this review, we aimed to gather lipidomic investigations in AAA and PAD. For that, English language publications were identified through a computerized search of PubMed database until January 2024, using the following keywords: “abdominal aortic aneurysm(s)” [MESH] OR “peripheral artery disease” [MESH] AND “lipidom*” OR “lipid profile” OR “phospholipid(s)” OR “fatty acid(s)” OR “sphingomyelin(s)” OR “ceramide(s)” OR (“spectrometry” AND “lipid”). A total of 14 papers (6 for AAA and 8 for PAD) were selected according to the eligibility criteria. Specifically, we focused on lipidomic research studies that used mass spectrometry (MS) approaches on any type of sample, excluding those that did not report the use of such approaches or were review papers. The data extracted from these studies were integrated to provide deeper insights into the pathogenesis of AAA and PAD.

### Lipidome profiling in AAA

To the best of our knowledge, only six studies [63–68] applied MS-based lipidomics to study AAA in clinical settings (Table 2). These studies applied an untargeted approach using either liquid chromatography coupled with mass spectrometry (LC–MS) techniques or imaging mass spectrometry (IMS) approaches using matrix-assisted laser desorption ionization (MALDI). Lipidomic analysis was done in samples from both human and murine models of the disease. Murine models of AAA have been used for many years; however, they do not fully replicate human disease. Still, mice models can reproduce inflammation, extracellular matrix destruction and aortic dilatation, all of which are observed in human aortic aneurysms. The angiotensin II (Ang II)-induced mouse AAA model in the atherosclerotic-susceptible strain (apolipoprotein E deficient (*ApoE*^*−/−*^)) is the most common model to study AAA due to its simplicity and similarity to the human disease. Males are more prone to develop the disease in the setting of mild hypertension with an enhanced incidence when hyperlipidaemia is promoted [69–72]. In this model, there is a stimulation of an inflammatory response, macrophage accumulation and thrombosis [73, 74]. Nonetheless, only two lipidomic studies on AAA reported the use of the murine model of this disease (although, comparing with human samples) [63, 64].

**Table 2:** Main lipid variations observed in murine models of AAA and in AAA human patients reported in published lipidomic studies, available in PubMed database, using MS approaches

Reference	Analytical method	Type of sample	Characteristics	Lipid extraction method	Results	
					Decreased	Increased
AAA in murine studies						
[63]	LC–MS/MS C18 column on a Thermo Q-Exactive	Plasma	10-wk-old male ApoE^−/−^ mice administered 1000 ng/kg per minute of Ang II by osmotic minipump for 4 wk	INF	LPC(15:0); LPC(16:0); LPC(O-18:0); LPC(18:1); LPC(20:0)	FA 20:4
[64]	LC–MS C18 column on a Q-TRAP	Tissue (AAA wall)	19- to 24-wk-old chow-fed male ApoE^−/−^ mice administered 1.1 mg·kg^−1^ *per* day of Ang II by osmotic minipump for 2 wk	MeOH/chloroform (2:1, v/v)	-	HODE; HDOHE; 5-HETE; 11-HETE; 12-HETE; 15-HETE from both PE and PC species OxPL with truncated PUFA
AAA in human studies						
[65]	LC–MS/MS C18 column on a Q-TRAP	Serum	161 AAA and 168 PAD (CT) Gender (M/F): 115/46 and 115/53 Age (years)^b^: 71.4 and 68.0	Chloroform/MeOH (2:1, v/v) + H_2_O/butanol + NH_4_COOH/MeOH	Sphingosine 1-phosphate	DG(18:0/18:2); DG(18:1/18:2); DG(18:2/18:2); TG(16:0/18:1/18:2); TG(16:0/18:2/18:2); TG(18:0/18:2/18:2); TG(18:1/18:1/18:2); TG(18:1/18:2/18:2); TG(18:2/18:2/18:2); TG(18:2/18:2/20:4);
[66]	MALDI-IMS with TOF and Q-TRAP	Tissue (AAA wall/aorta)	30 AAA and 7 CT Gender (M/F): 26/4 and 7/0 Age (years)^a^: 70.2 ± 9.0 and 66.1 ± 8.2	-	-	CE(18:1); CE(18:2); TG(52:2); TG(52:3); PC(16:0/18:0); PC(16:0/18:1)
[67]	LC–MS with Q-TOF	Tissue (AAA wall)	19 AAA and 9 CT Gender (M/F): 16/3 Age (years)^a^: 70.0 ± 5.2 (Clinical info of CT not found)	Chloroform/MeOH/HCl	*C16-ceramide* Negatively correlated with macrophages Positively correlated with T-cell infiltration *Sphingosine 1-phosphate* Positively correlated with neutrophils	
[68]	LC–MS with TOF LC–MS/MS with Q-TRAP	Tissue (AAA wall)	30 TAAA and 19 TNAA and 11 AAAA and 8 CT Gender (M/F): 28/2 and 13/6 and 8/3 and 7/1 Age (years)^a^: 72.0 ± 1.3 and 57.3 ± 3.5 and 67.9 ± 2.2 and 28.0 ± 4.2	Modified Bligh and Dyer + solid extraction	*TAAA* PE(P-38:5); PE(P-40:7); total PE-P *TAAA* + *TNAA* PE(P-38:6); DG(38:4); TG(48:1); TG(52:2); 14-HDoHE; 17-HDoHE; 15-HETE *AAAA* PE(P-36:5); PE(P-38:5); PE(P-38:6); total PE-P; SM(34:1); SM(34:1) + O	*TAAA* CE(18:2) + 2O; PC(34:2); PC(36:3); SM(34:0); SM(42:2); EPA; DHA; PGD_2_; 15-HETE; 15-HEPE; 17-HDoHE *TNAA* CE(18:1); CE(18:2); SM(34:2); SM(42:3) *TAAA* + *TNAA* LPC(16:0); LPC(18:0); GlcCer(34:1); GlcCer(42:1) *AAAA* TG(48:0); TG(50:0); 12-HETE; 12-HEPE; 10-HDoHE; 14-HDoHE; 20-HDoHE
[64]	LC–MS C18 column on a Q-TRAP	Tissue (AAA wall)	6 AAA Gender (M/F): 6/0 Age (years)^a^: 73.5 ± 8.4	MeOH/chloroform (2:1, v/v)	-	HODE; HDOHE; 5-HETE; 11-HETE; 12-HETE; 15-HETE from both PE and PC species OxPL with truncated PUFA
[63]	LC–MS/MS C18 column on a Thermo Q-Exactive	Plasma	70 AAA and 36 CT Gender (M/F): 61/9 and 33/3 Age (years)^a^: 70.4 ± 8.6 and 70.1 ± 8.7	INF	FA 18:2; LPC(15:0); LPC(16:0); LPC(16:1); LPC(17:0); LPC(18:0); LPC(P-18:0); LPC(18:1); LPC(18:2); LPC(20:4); LPC(22:6)	FA 20:4

*AAA* abdominal aortic aneurysm, *AAAA* abdominal atherosclerotic aortic aneurysm, *Ang II* angiotensin II, *ApoE*^*−/−*^ apolipoprotein E deficient, *CE* cholesteryl ester, *CT* control, *DG* diglycerides, *DHA* docosahexaenoic acid, *EPA* eicosapentaenoic acid, *FA* fatty acid, *GlcCer* glucosylceramide, *HCl* hydrochloric acid, *HDOHE* hydroxydocosahexanoic acid, *HETE* hydroxyeicosatetraenoic acid, *HEPE* hydroxyeicosapentaenoic acid, *HODE* hydroxyoctadecadienoic acid, *INF* information not found, *LC–MS* liquid chromatography-mass spectrometry, *LC–MS/MS* liquid chromatography-tandem mass spectrometry, *LPC* lysophosphatidylcholine, *MALDI-IMS* matrix-assisted laser desorption ionization-imaging mass spectrometry, *MeOH* methanol, *M/F* male/female, *OxPL* oxidized phospholipid, *PAD* peripheral artery disease, *PC* phosphatidylcholine, *PE* phosphatidylethanolamine, *PGD*_*2*_ prostaglandin D_2_, *PUFA* polyunsaturated fatty acid, *SM* sphingomyelin, *TAAA* thoracic atherosclerotic aortic aneurysm, *TG* triglycerides, *TNAA* thoracic nonatherosclerotic aortic aneurysm, *wk* week

^a^Values are expressed as mean ± standard deviation

^b^Values are expressed as mean

#### Lipidome profiling in murine models of AAA

A global lipidomic profiling of plasma samples from human AAA patients and ApoE^−/−^ mice (Ang II-driven disease model) was performed by Xie and co-workers [63] to better understand alterations in the lipid metabolism with the disease. It was determined that the main differences between AAA and control samples resided in the behaviour of lyso-PC (LPC) class species that were found to be significantly reduced in AAA. A special reference should be made to LPC(16:0) and LPC(18:0), which were found even more reduced in samples of ruptured AAA, compared with non-rupture AAA and controls, indicating a possible role as predictive biomarkers of aneurysm rupture. This study also revealed that plasma levels of LPC are inversely correlated with AAA diameter which might indicate that the biosynthesis of LPC may be impaired in AAA patients compared to controls [63]. The results of the murine model followed the same trend as the human cohort, with significantly decreased abundances of LPC in the mice administered with Ang II. LPC are well-established pro-inflammatory molecules linked to the development of atherosclerotic plaques [75] and endothelial cell dysfunction [76], hallmarks of AAA development. The increased levels of AA in both AAA patients and mice plasma are associated with an enhancement of the pro-inflammatory prostaglandin E2 formation and inhibition of leukotriene B4 synthesis [77].

Oxidized phospholipid (OxPL) species were detected in AAA lesions of aortic tissue from male ApoE^−/−^ mice (Ang II-driven disease model) (thrombus and AAA wall) [64]. The authors found that mice with Alox12^−/−^ and Alox15^−/−^ (murine isoforms of LOX) are able to generate smaller venous thrombi and bleed excessively when challenged, which were reverted by injecting OxPL into damaged tissue [78, 79]. However, they could not clearly define which OxPL molecular species are formed upon clotting, or the predominant forms that contribute to haemostasis/thrombosis: it was not reported how OxPL may interact with the coagulation factors and influence vascular inflammation. Additionally, the procoagulant role of the surface of circulating blood cells, required for haemostasis, has not been investigated in the context of human AAA. With that in mind, in this study, the authors also evaluated the lipidome of aortic tissue from humans (undergoing open AAA repair) and detected, in both human and mice samples, a significant number of OxPL species belonging to the classes of phosphatidylethanolamine (PE) and phosphatidylcholine (PC), namely hydroxyoctadecadienoic acid (HODE), hydroxydocosahexanoic acid (HDOHE) and 5-, 11-, 12- and 15-hydroxyeicosatetraenoic acid (HETE)-PL [64]. These oxidized species were not found in the aortic wall from control samples. Additionally, OxPL containing truncated PUFA were found in AAA wall and thrombi. It is most likely that OxPL deposit on the surface of the vessel wall, providing a localized surface to enable coagulation factor binding and activation. Furthermore, Alox12 and Alox15 gene products (12-LOX and 12/15-LOX) generate similar OxPL isomers in mice, specifically the abundant 12-HETE-PLs, which is in agreement with the findings of this study, suggesting these are the most likely candidates for driving AAA in the vessel wall [80–82]. OxPL have also been considered pro-inflammatory agents and can contribute to aggravate AAA [58, 83, 84]. Thus, this study shows that OxPL may play an important role in the development of thrombus and AAA due to their presence in both angiotensin II/ApoE mice model and human AAA tissue [64].

#### Lipidome profiling in human studies of AAA

AAA-associated lipid species were assessed on serum samples of AAA patients by Moxon and partners [65]. The study compared AAA patients with individuals with PAD as controls. The results showed that sphingosine 1-phosphate was significantly decreased in AAA samples compared with PAD, revealing a negative association with AAA. These results are in agreement with the findings of previous studies [85]. On the other side, AAA presented significantly higher levels of three diglycerides (DG) and seven triglycerides (TG) species, all bearing fatty acid 18:2, which were found to be positively correlated with the presence of AAA. Increased circulating TG may contribute to endothelial dysfunction through generation of ROS in patients with diabetes mellitus [86]; however, TG do not directly participate in vascular damage in PAD.

Human tissue from the aneurysmal wall was examined by MALDI imaging by Tanaka et al. [66]*.* to clarify the role of lipids in the pathobiology of AAA. The study revealed significantly high intensities of cholesteryl esters (CE) CE(18:1), CE(18:2), TG(52:2), TG(52:3), PC(16:0/18:0) and PC(16:0/18:1) in the aneurysmal tissue of AAA patients compared with the non-aneurysmal one. As expected, CE were identified around atherosclerotic legions. Additionally, it was found that the distribution of TG in the aneurysmal wall was similar to the distribution found in cells with morphological characteristics similar to adipocytes, revealing the potential role of adipocytes in the reduction of aortic wall strength. Thus, the authors hypothesize that local adipocytes may suffer from hypertrophy due to changes in the TG levels in tissues located in the abdominal aorta [66]. In this sense, the hypertrophic adipocytes may influence the progression of AAA.

AAA is abundantly surrounded by perivascular adipose tissue (PVAT), and its sphingolipid profile was analyzed by Folkesson and colleagues [67]. The study intended to establish a correlation between sphingolipid metabolites and pro-inflammatory factors in AAA. It was determined that C16-ceramides were strongly negatively correlated with macrophages but positively correlated with T-cell infiltration in PVAT of AAA. Moreover, sphingosine 1-phosphate was found to be positively associated with neutrophils in PVAT. It has been described that the abnormal vessel wall of AAA contains abundant immune cells [87, 88], and the results of this study serve to show that some sphingolipid species are directly associated with the pro-inflammatory state of this pathology [67].

The lipidomic signatures of the aortic media from patients with thoracic nonatherosclerotic aortic aneurysm (TNAA), thoracic atherosclerotic aortic aneurysm (TAAA) and abdominal atherosclerotic aortic aneurysm (AAAA) were determined by Saito and co-workers [68]. The study showed several findings worth mentioning. Considering TNAA, it was observed lower levels of plasmalogen PE, TG, 12- and 15-LOX metabolites and increased levels of LPC, glucosylceramides (GlcCer), prostaglandin PGD_2_ and 5-LOX metabolites during the development of thoracic aortic aneurysms. In TAAA, there was a significant decrease of plasmalogen PE and an increase in PC, sphingomyelin (SM), CE and TG species and prostaglandin PGD_2_ and 15-LOX metabolites. Regarding AAA, it was also found a reduction of plasmalogen PE and an increase of 12-LOX metabolites. LOX metabolites are usually pro-inflammatory mediators contributing to the increase of ROS generation via NADPH oxidase stimulation [89]. Plasmalogen PE are well-known endogenous antioxidants, and their reduction in the aortic media of both TAAA and AAAA may have a pivotal role in atherosclerotic aortic aneurysm development [68]. The authors concluded that the reduction of plasmalogen PE species may be associated with lower antioxidant effects contributing to enhance the oxidative stress environment generated during atherosclerotic events, thus aggravating these conditions. Additionally, the increase of prostaglandin PGD_2_ in the aortic media may counteract TAAA development since it has been found that the inhibition of lipocalin-type PGD_2_ synthase accelerates aortic lipid accumulation and development of atherosclerosis in murine models [90].

Overall, the studies previously mentioned suggest that the alterations in lipid metabolism that occur during AAA can be evaluated through plasma/tissue lipidomic analysis. Phospholipids, TG, oxylipins and products of lipid oxidation were found to be significantly modified in this disease, confirming the high impact of oxidative stress in the pathogenesis of AAA. Furthermore, besides the already reported lipid peroxidation products like MDA and 4-HNE, oxidized phospholipids may play important roles in haemostasis and vascular cell behaviour.

### Lipidome profiling in PAD

Up to date and to the best of our knowledge, lipidomic analysis on PAD [91–98] was performed using gas chromatography-mass spectrometry (GC–MS) for fatty acids profiling (FA), using MALDI imaging to visualize individual molecules on tissue sections and LC–MS techniques for lipidomic signatures (Table 3). Lipidomic analysis was performed also on both murine and human samples of PAD. The human studies gathered in this review report lipidomic analysis on different types of samples.

**Table 3:** Main lipid variations observed in murine models of PAD and in PAD human patients reported in published lipidomic studies, available in PubMed database, using MS approaches

Reference	Analytical method	Type of sample	Characteristics	Lipid extraction method	Results	
					Decreased	Increased
PAD in murine studies						
[95]	MALDI with Q-TRAP	Tissue (aortic roots)	12- and 60-wk-old male ApoE^−/−^ mice with a C57BL/6 genetic background	-	-	CE(18:1); CE(18:2); PC(16:0/20:4); PC(18:0/20:4)
[98]	MALDI with orbitrap	Tissue (aorta and carotid)	14- to 60-wk-old male ApoE^−/−^ mice	-	-	7 ketocholesterol; CE(18:2); CE(20:4); LPE(22.0); LPC(18:2)
PAD in human studies						
[93]	GC–MS	Plasma	113 PAD and 122 CT Gender (M/F): 57/56 and 63/59 Age (years)^b^: 67.3 and 67.4	INF	TG fraction: AA CE fraction: EPA; DHA; EPA/AA ratio PL fraction: EPA; DPA; DHA	-
[95]	MALDI with Q-TRAP	Perivascular adipose tissue	INF	-	-	CE(18:1); CE(18:2); PC(16:0/20:4); PC(18:0/20:4); TG(18:0/18:1/18:2)
[94]	GC–MS	Serum	101 PAD + 373 CT Gender (M/F): 81/20 and 306/67 Age (years)^a^: 73.2 ± 0.9 and 72.4 ± 0.4	Folch	FA 18:3 *n*−6; EPA; DPA; DHA; EPA/AA ratio; DHA/AA ratio	-
[96]	GC–MS	Plasma	98 PAD + 20 CT Gender (M/F): 73/25 and 11/9 Age info available at [96]	Chloroform/MeOH (2:1, v/v) + solid phase extraction	-	16-HETE with CVA PGE_2_ with angina TXB_2_/6KPGF_1α_ with TIA 8,9-DiHETRE with ACS
[92]	GC–MS	Adipose tissue	870 PAD Gender (M/F): 537/333 Age (years)^b^: 58.6	INF	EPA; DHA; EPA + DHA	DPA
[91]	GC–MS	Faeces	53 PAD with diabetes Gender (M/F): 30/23 Age (years)^a^: 59.2 ± 10.3	H_2_O/isopropanol/HCl	-	Acetate; propionate; butyrate; valerate; total short-chain FA
[98]	MALDI with orbitrap	Tissue (peripheral artery)	8 atherosclerosis + 3 CT Gender (M/F): 5/3 and 0/3 Age (years)^a^: 63 ± 14.8 and 63 ± 5.4	-	-	Ester cholesteryl acetate; LPE(18:0); LPC(16:1); LPC(22:5); LPC(22:6); 16:0 Glc-cholesterol; 16:3 Glc-cholesterol; 18:3 Glc-cholesterol; 22:0 Glc-cholesterol
[97]	LC–MS C18 column on a QQQ-TRAP	Plasma	98 PAD + 20 CT Gender (M/F): 72/26 and 11/9 Age info available at [97] for patients and [114] for CT	Solid phase extraction	-	With tobacco smoking 12,13-EpODE; 12,13-EpOME; 13-HODE; 13-OXoODE; 9-HODE; 9,10-DiHOME; KODA-PPC

*AA* arachidonic acid, *ACS* acute coronary syndrome, *ApoE*^*−/−*^ apolipoprotein E deficient, *CE* cholesteryl ester, *CT* control, *CVA* cerebrovascular accidents, *DHA* docosahexaenoic acid, *DiHETRE* dihydroxyeicosatrienoic acid, *DiHOME* dihydroxyoctadecenoic acid, *DPA* docosapentaenoic acid, *EPA* eicosapentaenoic acid, *EpODE* epoxy-octadecadienoic acid, *EpOME* epoxyoctadecenoic acid, *FA* fatty acid, *GC–MS* gas chromatography-mass spectrometry, *Glc* glucosyl, *HCl* hydrochloric acid, *HETE* hydroxyeicosatetraenoic acid, *HODE* hydroxyoctadecadienoic acid, *INF* information not found, *LC–MS* liquid chromatography-mass spectrometry, *LPC* lysophosphatidylcholine, *LPE* lysophosphatidylethanolamine, *MALDI* matrix-assisted laser desorption ionization, *MeOH* methanol, *M/F* male/female, *PAD* peripheral artery disease, *PC* phosphatidylcholine, *PGE*_*2*_ prostaglandin E2, *PL* phospholipid, *TG* triglycerides, *TIA* transient ischemic attacks, *TXB*_*2*_*/6KPGF*_*1α*_ thromboxane B_2_/6 keto prostaglandin F_1α_, *OXoODE* oxooctadecadienoic acid, *wk* week

^a^Values are expressed as mean ± standard deviation

^b^Values are expressed as mean

#### Lipidome profiling in murine models of PAD

Identification and visualization of specific lipid markers for aortic atherosclerotic lesions were made by Zaima and partners [95]. The study analyzed tissue from aortic roots of ApoE^−/−^ mice (C57BL/6 genetic background) by IMS and identified CE(18:1), CE(18:2), PC(16:0/20:4) and PC(18:0/20:4) to be significantly increased in the atherosclerotic aortic tissue. These species were not found in nonatherosclerotic lesions, revealing an atherosclerotic site-specificity and their potential undesirable role in the formation of atherosclerotic plaques. The authors also collected perivascular adipose tissue from human patients with PAD and performed the same analysis, determining the increase of the same species found for mice samples, plus the lipid species TG(18:0/18:1/18:2). Even though TG involvement in the development of atherosclerosis remains unknown, this lipid class may play an important role in the evolution of atherosclerosis since TG deposits were found in aortic atherosclerotic lesions, while cholesterol levels were within the normal range [99].

A direct comparison of murine and human atherosclerotic plaques was performed by Khamehgir-Silz et al*.* to determine lipid markers able to differentiate disease progression and medication [98]. The aortic tissue of ApoE^−/−^ mice was initially studied to identify possible lipid markers for the subsequent evaluation of human atherosclerotic vessel samples. The study found that the levels of 7 ketocholesterol CE(18:2), CE(20:4), LPC(18:2) and lyso-PE (LPE(22.0)) were significant to differentiate murine atherosclerotic tissue from the nonatherosclerotic one, being identified as ApoE-specific plaque biomarkers. Then, in the analysis of atherosclerotic vessel samples from PAD patients, the levels of cholesteryl acetate, LPE(18:0), LPC(16:1), LPC(22:5), LPC(22:6) and the glucosylated cholesterol species (Glc-cholesterol) esterified with 16:0, 16:3, 18:3 and 22:0 were identified as significant human atherosclerotic markers due to their increased abundances [98]. The dissimilarity of the markers found for mice and human samples may be explained by the fact that human vascular specimens present a more diverse patient-dependent lipid distribution, which differ from the vascular specimens derived from the monogenetic ApoE^−/−^ mice. This could also be explained by the different stages of atherosclerosis progression that reflect in different lipid profiles. Additionally, diet, lifestyle, smoking habits [100] as well medication of the patients with cholesterol-lowering drugs [101] influence the lipid profile.

#### Lipidome profiling in human studies of PAD

The variation of the FA profile of the plasma of PAD patients, compared with non-disease condition, was reported by Leng and collaborators. The FA profile of the TG, PL and CE classes separated by thin-layer chromatography showed differences in the disease state [93]. In the TG fraction, it was found significantly decreased levels of AA. Eicosapentaenoic acid (C20:5, EPA) and docosahexaenoic acid (C22:6, DHA) were significantly reduced in the PL and CE fractions. The ratio between EPA and AA (EPA/AA ratio) was markedly decreased in the CE fraction. Additionally, docosapentaenoic acid (C22:5, DPA) showed considerably lower levels in the PL fraction. EPA competes with AA for COX activity, leading to the formation of different prostacyclins and thromboxanes that contribute to a more vasodilatory state with lower platelet aggregation [102]. However, the results of this study regarding a lower EPA/AA ratio in the CE fraction of PAD patients suggest that EPA is being either less produced or more oxidized in PAD, possibly due to the increased inflammatory state.

The FA alterations in the sera of PAD patients were investigated by Gautam and colleagues [94]. The results showed significantly reduced levels of C18:3 *n*−6, EPA, DPA, DHA, EPA/AA ratio and DHA/AA ratio in PAD, in accordance with the study of Leng et al*.* [93]. It was also shown that the low levels of C18:3 *n*−6 and EPA:AA ratio were significantly associated with the presence and advanced status of the disease [94]. Studies with C18:3 *n*−6 supplementation support an anti-inflammatory or immunomodulatory role of C18:3 *n*−6 [103, 104], a vasodilatory [105], blood pressure-lowering [106] and even an inhibitory effect on smooth muscle cell proliferation associated with the progression of atherosclerosis. Thus, the authors suggest that C18:3 *n*−6 may have a potential role in inflammation mediating the development of peripheral atherosclerosis.

Connecting PAD with other CVD, the study conducted by Caligiuri et al*.* aimed to show that higher levels of eicosanoids derived from *n*−6 FA increase the probability of cardiovascular and cerebrovascular events in patients with PAD [96]. They analyzed plasma samples of PAD patients who have stable angina, acute coronary syndrome (ACS), transient ischemic attacks (TIA) and cerebrovascular accidents (CVA) and assessed their relationship with plasma FA and oxylipin concentrations. The authors found that the levels of four plasma oxylipins were changed in the presence of an event. Plasma 16-hydroxyeicosatetraenoic acid (16-HETE), thromboxane B_2_/6 keto prostaglandin F_1α_ (TXB_2_/6KPGF_1α_) ratio and prostaglandin E_2_ (PGE_2_) were markedly higher in PAD patients with CVA, TIA and angina, respectively. Also, 8,9-dihydroxyeicosatrienoic acid (8,9-DiHETRE) was positively correlated with ACS in PAD individuals. This study identified specific inflammatory oxylipins that may be considered potential markers/therapeutic targets of cardiovascular/cerebrovascular events [96].

The correlations between the content of marine *n*−3 PUFA in adipose tissue and the risk of PAD incidence were also evaluated [92]. It was found that PAD patients had lower levels of EPA, DHA and EPA + DHA and higher levels of DPA. The study showed an inverse association between the marine *n*−3 PUFA, EPA and DHA, as well as between the combined content of EPA + DHA in adipose tissue and the risk of PAD. Marine *n*−3 PUFA were found to have a protective role against atherosclerotic diseases [107–109]; thus, the results of this study suggest that increased levels of marine *n*−3 PUFA are associated with a lower risk of developing the disease [92].

As PAD is a very common comorbidity of diabetes mellitus, Muradi and his research team investigated the relationship between short-chain FA (SCFA) levels and PAD in faecal samples of patients with diabetes [91]. SCFA are produced in the gut microbiota and are known to inhibit cholesterol synthesis and the inflammatory process of atherosclerosis, thereby preventing immune cells from migrating, proliferating and producing a variety of cytokines [110]. Additionally, some studies have discovered an inverse relationship between SCFA and the size of atherosclerotic plaques [111]. In Muradi’s study, it was reported increased levels of acetate, propionate, butyrate, valerate and total SCFA in PAD patients with diabetes. It was also shown that there is a significant positive correlation between the levels of propionate, butyrate, total SCFA and blood glucose. The SCFA acetate, propionate and valerate presented likewise a significant positive correlation with the diameter of the superficial femoral artery and dorsal pedis artery and with the peak systolic velocity of the posterior tibial, popliteal, common femoral and superficial femoral arteries. On the other side, valerate revealed an inverse correlation with TG levels [91]. Acetate is known to inhibit the production of oxalate, an atherogenic mediator that increases lipid oxidation and, thus, decreases the risk of fat accumulation in peripheral arteries [112].

The plasma oxylipidome, including oxylipins and oxidized PC, was characterized by Caligiuri and colleagues from PAD patients with a history of tobacco smoking [97]. The study showed that PAD patients who never smoked had a significantly higher level of total circulating plasma oxylipins than those with a past history of smoking or currently smoking, in particular, the linoleic acid-derived oxylipins 12,13-epoxy-octadecadienoic acid (12,13-EpODE), 12,13-epoxyoctadecenoic acid (12,13-EpOME), 13-hydroxyoctadecadienoic acid (13-HODE), 13-oxooctadecadienoic acid (13-OXoODE), 9-hydroxyoctadecadienoic acid (9-HODE) and 9,10-dihydroxyoctadecenoic acid (9,10-DiHOME). The authors suggest that PAD individuals with no smoking history may (i) enzymatically produce more oxylipins from PUFA, (ii) exhibit less degradation/elimination of oxylipins or (iii) exhibit a greater release of oxylipins to the plasma. Oxidized PC were also analyzed, and it was found that KODA-PPC plasma was significantly increased in the smoking group *versus* never or past-smoking individuals, which may reflect a potentially significant role of oxidized PC in the pathophysiology of PAD in the absence of tobacco consumption. The results reported in this research reveal the contrasting effect between the elevated levels of total oxylipins and pro-atherogenic oxidized PC in PAD patients with no smoking history [97].

In summary, lipidomic findings on PAD revealed alterations in the FA metabolism, especially involving *n*−3 and *n*−6 PUFA. The *n*−3 PUFA are known to lower plasma TG, impair platelet reactivity, slightly lower blood pressure and may stabilize atherosclerotic plaques [113]. *n*−3 PUFA also possess anti-inflammatory properties, in part by generating less pro-inflammatory eicosanoids than the more commonly eaten *n*−6 PUFA; thus, *n*−3 and *n*−6 PUFA may play an important role in the pathophysiology of PAD. Additionally, oxylipins are revealing a promising marker role for lipid alterations induced by cardio/cerebrovascular events and smoking habits. Nonetheless, the lack of studies in the same sample type impairs the establishment of a well-defined lipid profile of PAD, revealing the need for more investigations in each matrix (Table 3).

**Fig. 1:**
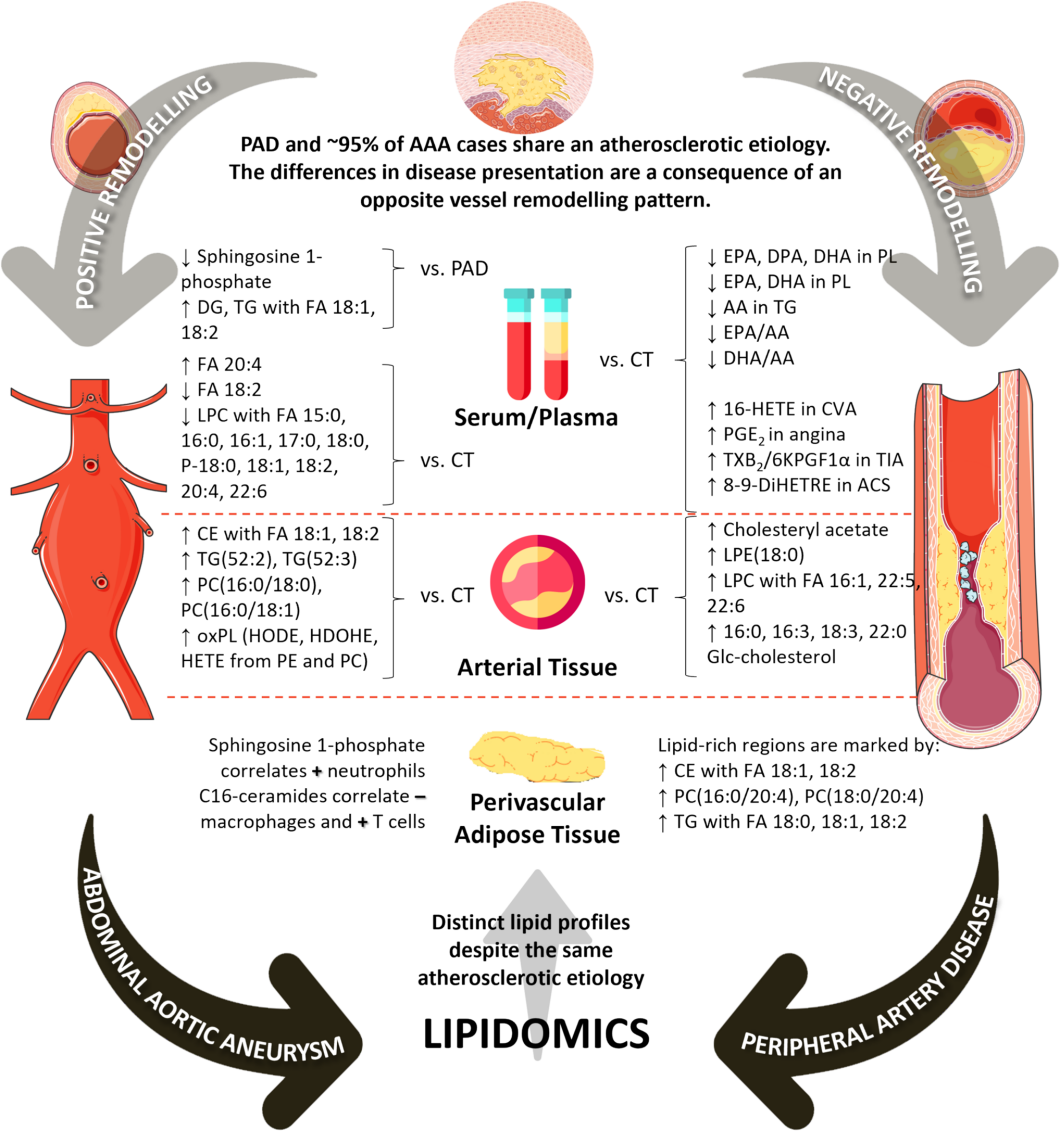
Lipidomics evidences a distinct lipid profile between abdominal aortic aneurysm and peripheral artery disease *in situ* (arterial tissue), in perivascular adipose tissue and in circulation, despite the same atherosclerotic etiopathogenesis [116, 117]. Only studies in humans were considered. All AAA cases were of atherosclerotic origin (95% of the cases). Some graphical elements were adapted from Flaticon and Servier Medical Art. Abbreviations: AA, arachidonic acid; AAA, abdominal aortic aneurysm; ACS, acute coronary syndrome; CE, cholesteryl ester; CT, control; CVA, cerebrovascular accidents; DG, diglycerides; DHA, docosahexaenoic acid; DiHETRE, dihydroxyeicosatrienoic acid; DPA, docosapentaenoic acid; EPA, eicosapentaenoic acid; FA, fatty acid; Glc, glucosyl; HDOHE, hydroxydocosahexanoic acid; HETE, hydroxyeicosatetraenoic acid; HODE, hydroxyoctadecadienoic acid; LPC, lysophosphatidylcholine; LPE, lysophosphatidylethanolamine; oxPL, oxidized phospholipids; PAD, peripheral artery disease; PC, phosphatidylcholine; PE, phosphatidylethanolamine; PGE_2_, prostaglandin E_2_; PL, phospholipid; TG, triglycerides; TIA, transient ischemic attacks; TXB_2_/6KPGF_1α_, thromboxane B_2_/6 keto prostaglandin F_1α_

Although being very few, these studies clearly demonstrate the role of lipids in inflammatory/oxidative processes, in atherosclerosis and in vascular remodelling (Fig. 1). Imaging diagnostic tools like ultrasound, CTA and angiography are already well-established techniques in the management of both AAA and PAD. Clinical lipidomics has also potential as a tool to aid AAA and PAD in both diagnosis, risk monitoring and therapeutics. For now, however, cholesterol, in the form of LDL-C, is the only lipid biomarker integrated in clinical decision-making. For instance, in the 2024 ACC/AHA/AACVPR/APMA/ABC/SCAI/SVM/SVN/SVS/SIR/VESS guidelines for the management of lower extremity PAD, patients on maximally tolerated statin therapy with an LDL-C level ≥ 70 mg/dL are recommended to be prescribed an additional lipid-lowering drug to reduce the risk of progression to more symptomatic or limb-threatening PAD [115]. Similarly, in the ESVS 2024 Clinical Practice Guidelines on the Management of AAA, it is recommended to reduce the cardiovascular risk of patients with AAA, by, among others, reducing LDL-C to < 70 mg/dL with an aggressive statin therapy regimen [10]. Apart from cholesterol, other lipid biomarkers may improve the clinical decision-making process. Lipidomic analysis of AAA and PAD patients in different stages of disease progression can be the key to find those biomarkers, which will improve the efficiency of clinical practice by stratifying patients for interventions, predicting aneurysm rupture risk or limb ischemia and guiding surgical timing. Still, more studies are required since the ones published so far only concern phospholipids and FA and were not even driven from the same type of samples. In fact, the lipidome includes many other classes that have also been reported as changed in CVD, such as sphingomyelins, ceramides and cholesteryl esters [2], whose potential as clinical biomarkers remains elusive. The analysis of plasma/serum, aortic wall tissue, adipose tissue and faeces reveals different information according to the matrix. It would be beneficial to have more studies on each biofluid/product to gain a broader knowledge of the lipid alterations that occur in both diseases and the correlations that may exist with their pathophysiology.

The application of machine learning to integrate lipidomics and phenomics data can be useful to improve the monitorization and treatment strategies established for both AAA and PAD. Machine learning relies on statistical algorithms and techniques that can model large datasets and detect useful patterns. The application of the prediction models determined by machine learning in medicine could be beneficial for a better stratification and risk assessment of patients eligible for pharmacological or vascular surgical interventions [118]. The incorporation of lipidomic data would also complement existing risk stratification tools as recently reported for PAD patients where risk stratification was further improved by adding lipidomic variables to the model. Serum levels of ether-linked PC species were negatively associated with incident myocardial infarction, showing potential to be considered prognostic biomarkers in at-risk populations [119]. Moreover, elevated levels of serum-free fatty acids were also associated with increased probability of clinical PAD over long-term follow-up [120]. Overall, AAA and PAD could benefit from the improvement of machine learning strategies with the integration of lipidomic data; however, the research in this area is still in its infancy.

## Concluding remarks and future perspectives

AAA and PAD are two diseases for which there is no precise monitoring of the disease onset and progression. AAA and PAD are multifactorial diseases; thus, a multimarker approach would be more suitable to face the challenges of diagnostics and prognostics. Very few lipidomic studies report alterations in the lipid signature of AAA and PAD patients, either in PL or in the FA metabolism, respectively. Although AAA and PAD have a common atherosclerotic root, lipidomics demonstrates the existence of distinct lipid profiles; thus, there is a clear and urgent need to develop more research in this area regarding these silent diseases. Future studies that would greatly improve the scientific knowledge on AAA and PAD involve carrying out (i) a cross-sectional analysis of tissue samples and their relationship with secondary risks and major adverse cardiovascular events and (ii) a prospective study of the lipid species in circulation to develop predictive models of AAA and PAD (such as incidence, progression and therapy effectiveness). The incorporation of the knowledge gained through lipidomics into machine learning methods would greatly improve precision medicine, allowing not only an adequate monitoring of AAA and PAD progression but also a more personalized pharmacological or surgical treatment.

## Data Availability

Not applicable.
